# Adults with type 1 diabetes who sleep 7–9 hours per night present lower glycemic variability: a cross-sectional study

**DOI:** 10.3389/fendo.2025.1745272

**Published:** 2026-01-12

**Authors:** Anna Duda-Sobczak, Michal Kulecki, Stanislaw Pilacinski, Dariusz Naskret, Dorota Zozulinska-Ziolkiewicz

**Affiliations:** 1 Poznan University of Medical Sciences, Department of Internal Medicine and Diabetology, Poznan, Poland; 2 Poznan University of Medical Sciences, Doctoral School, Poznan, Poland

**Keywords:** continuous glucose monitoring, glycemic variability, sleep duration, sleep quality, type 1 diabetes

## Abstract

**Introduction:**

The National Sleep Foundation (NSF) recommends 7–9 hours of sleep per night for adults. Inadequate sleep may negatively impact the outcomes of diabetes treatment.

**Objectives:**

This study aimed to investigate the associations between sleep duration and quality and glycemic variability in adults with type 1 diabetes.

**Patients and methods:**

155 participants with type 1 diabetes (73 men, 47%), mean (SD) age 33 (9) years, median (IQR) diabetes duration 12 (8-20) years, completed the Pittsburgh Sleep Quality Index (PSQI) questionnaire. Continuous glucose monitoring (CGM) data were analyzed using Glyculator 3.0. The ANOVA/Kruskal-Wallis test with *post-hoc* Bonferroni correction analysis, logistic regression, and multivariable linear regression models were used.

**Results:**

78 participants (50.3%) met the NSF criteria of recommended sleep duration, 56 (36.1%) declared sleeping less than 7h, and 21 (13.6%) sleeping more than 9h. Compared with participants sleeping 7-9h per night, each other group had significantly higher: mean glucose, coefficient of glycemic variability (CV), glycemia risk index (GRI), high blood glucose index (HBGI), mean amplitude of glucose excursions (MAGE), glycemic risk assessment in diabetes equation (GRADE), mean of daily differences (MODD) and lower time-in-range (TIR). No differences in sleep quality, low blood glucose index (LBGI), HbA1c, or diabetes duration were shown among groups. In multivariable logistic regression analysis sleeping 7-9h per night was associated with lower CV, MAGE and MODD after adjustment for age, sex and HbA1c.

**Conclusions:**

Adults with type 1 diabetes who sleep 7–9 hours per night present lower glycemic variability compared with those sleeping less or more.

## 1 Introduction

Healthy sleep, proper diet, regular physical activity, and social interactions are essential for physiological and psychological well-being and help maintain metabolic homeostasis. Impaired sleep has been linked to many chronic conditions contributing to significant public health burdens, including cardiovascular disease (1), cognitive decline and dementia (2), mental health issues (3), disruptions in glucose metabolism and insulin resistance (4). Humans spend approximately one-third of their lives asleep. Nowadays, sleep deficiency affects 20–45% of the general population, and its prevalence is rising due to factors such as sedentary work habits, increased use of electronic devices, and mistimed food intake (5, 6). Adequate sleep comprises both its duration and quality. Appropriate sleep duration ranges vary throughout the life span, and the National Sleep Foundation recommends 7-9h of sleep per night for young adults (18–25 years) and adults (26–64 years), with consistent onset and offset of sleep timing (7, 8). Worldwide, approximately 537 million people aged 20–79 years have diabetes (9). If not properly treated, diabetes can lead to complications such as cardiovascular disease and microvascular damage (10). People with diabetes across the lifespan often experience sleep disruptions and reduced sleep quality, which may interfere with achieving and maintaining target glucose levels (11, 12). Recently, sleep has emerged—alongside diet and physical activity—as a modifiable lifestyle factor in the non-pharmacological management of diabetes. The American Diabetes Association has identified sleep as an important issue in managing diabetes and recommends “the assessment of sleep pattern and duration as part of the comprehensive medical evaluation based on emerging evidence suggesting a relationship between sleep quality and glycemic control (13). Although type 1 diabetes is less prevalent than type 2 and accounts for approximately 10% of diabetes, the incidence of type 1 diabetes has risen globally in recent decades, with the current rate of 15 per 100,000 people and prevalence of 9.5 per 10,000 people with significant differences in incidence rates among countries worldwide (14). Type 1 diabetes is an autoimmune disease that results in destruction of pancreatic β cells and leads to insulin deficiency, necessitating exogenous insulin administration to regulate blood glucose levels. Poor metabolic control of type 1 diabetes can lead to neurovascular complications (i.e., nephropathy, retinopathy, and neuropathy), cardiovascular disease, and premature mortality.

Despite the abundant evidence linking sleep deficiencies and type 2 diabetes, far less research has focused on people with type 1 diabetes.

The study aimed to assess the relationship between sleep duration and quality and glycemic variability in adults with type 1 diabetes.

## 2 Materials and methods

The study enrolled 217 eligible participants with type 1 diabetes, who were under the control of the outpatient unit of the Department of Internal Medicine and Diabetology, Poznan University of Medical Sciences, an academic referral center for diabetes care in western Poland. This study adhered to the ethical guidelines set by the local Ethical Committee (approval No 106/24, 8^th^ February 2024) and followed the principles of the Declaration of Helsinki. During the appointed regular check-outs, participants were enrolled in the study throughout the year 2024. All participants were provided with a comprehensive written and verbal description of the study before engaging in any study-related activities. Informed consent was voluntarily obtained prior to participation.

### 2.1 Inclusion and exclusion criteria

The main study inclusion criteria were:

- Diagnosis of type 1 diabetes for at least 1 year
- Age ≥ 18 years
- Written informed consent and adherence to the study protocol
- Continuous glucose monitoring (CGM) use for at least 6 months

**Figure 1:**
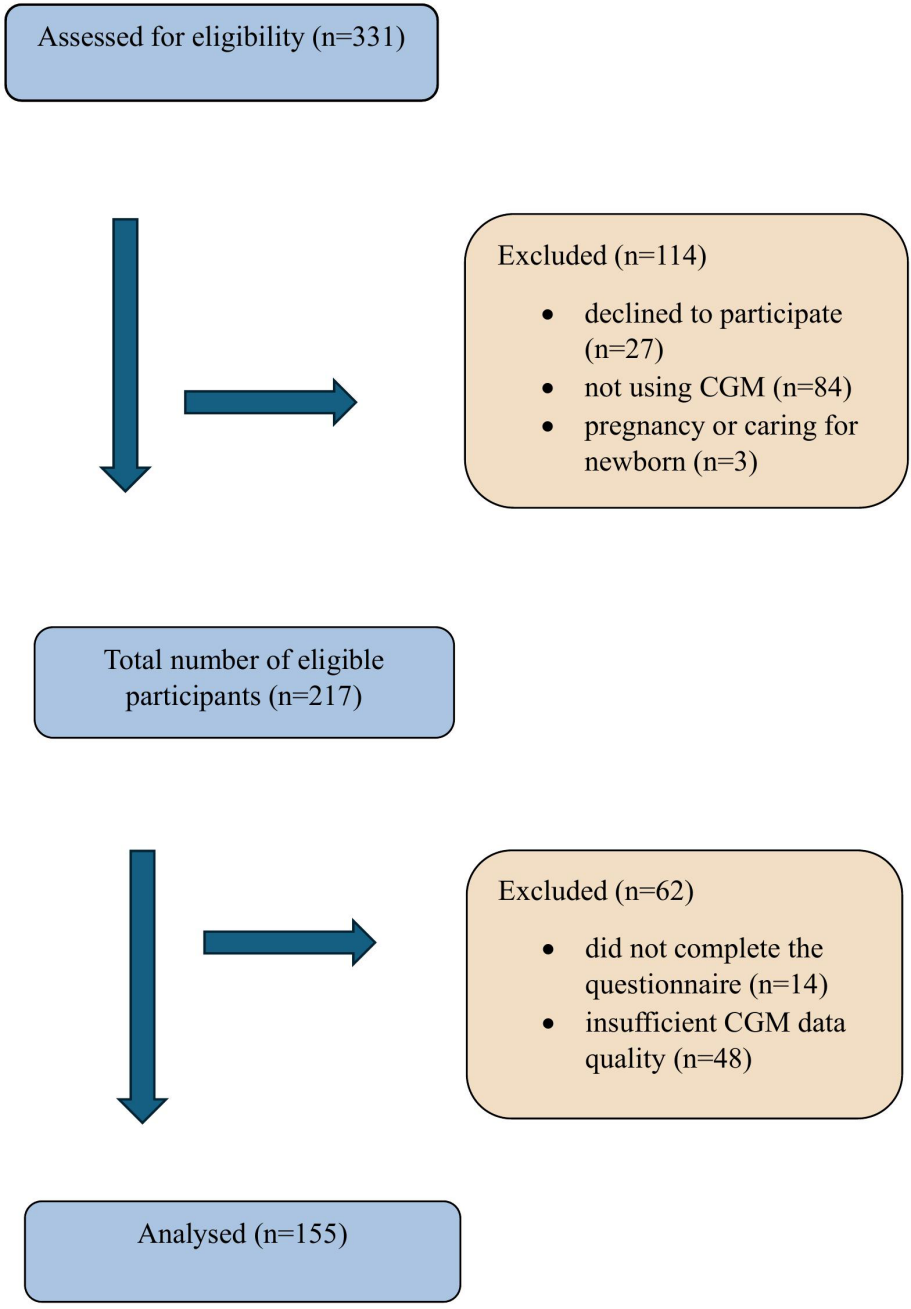
Study flowchart.

The exclusion criteria were as follows:

- Pregnancy or taking care of a newborn/baby
- Use of medications significantly affecting sleep
- Evidence of alcohol or drug abuse
- Intensive care unit admission within the past month
- Sleep apnea syndrome,
- Shift work;

Of 217 eligible participants, 48 were excluded due to insufficient quality of CGM data, and 14 denied completing the questionnaire. Analyses were made based on the data from 155 participants with type 1 diabetes (73 men, 47%). The mean (SD) age of participants was 33 (9) years, median diabetes duration was 12 (IQR 8-20) years and mean (SD) HbA_1c_ was 57.2 (11.3) mmol/mol (7.4 [1.0]%). The study flowchart is shown in **Figure 1**.

### 2.2 Procedures

Participants underwent clinical examination, including body weight and height, and body mass index BMI was calculated. Clinical data, including duration of diabetes, diagnosed chronic complications of diabetes, and drinking alcohol, were retrieved from both the electronic hospital records and the interview with the participant. All participants were asked to complete the Pittsburgh Sleep Quality Index (PSQI) via a survey link sent via email. The responses were collected and stored in REDcap, a secure web application for building and managing online surveys and databases. The Pittsburgh Sleep Quality Index (PSQI) is a 19-item self-report questionnaire assessing sleep duration and quality during the preceding month (15). The 19 questions are combined into 7 clinically-derived component scores, each weighted equally from 0–3. The 7 component scores are added to obtain a global score ranging from 0–21, with higher scores indicating worse sleep quality. The clinical and psychometric properties of the PSQI have been formally evaluated by several research groups. PSQI examines seven components: sleep quality, latency, habitual sleep efficiency, sleep duration, sleep disturbances, use of sleep medication, and daytime dysfunction. Self-reported sleep quality was categorized as good or poor according to the cutoff of the original questionnaire (PSQI score >5) (16). The PSQI demonstrates a sensitivity of 89.6% and specificity of 86.5% for identifying cases with sleep disorder, using a cut-off score of 5. Validity is further supported by similar differences between groups using PSQI or polysomnographic sleep measures.

### 2.3 Parameters of glycemic variability

All patients were treated with intensive functional insulin therapy, in which the insulin dose was adjusted based on blood glucose levels and the carbohydrate content of the meal (multiple daily injections, n=83, insulin pump, n=72). The devices used for continuous glucose monitoring (CGM) included Dexcom G7 or One+ (n=10, Dexcom, San Diego, USA), FreeStyle Libre (n=132, Abbott Diabetes Care, Alameda, USA), Medtronic Guardian G3 or G4 (n=13, Medtronic MiniMed, Inc., Northridge, CA, USA). CGM data, registered simultaneously, were downloaded and analyzed for all eligible participants using Glyculator 3.0 software (17). GlyCulator 3.0 supports cross-platform CGM data analysis from different sensor providers in a unified format.

All of the sensors were equipped with alarms for hypoglycemia and hyperglycemia, with threshold values individually set by each user. Since the PSQI addresses the preceding month, we analyzed the data covering 30 days of 24-hour continuous glucose monitoring, counting backward from the date of completing the survey. There are several glycemic variability (GV) metrics that can be derived from raw CGM data. Time in range (TIR), defined as the percentage of time spent in the target glucose range (70-180mg/dl), is the most recognizable and commonly used in clinical practice. The American Diabetes Association and the European Association for the Study of Diabetes recommend achieving >70% of time in the target glucose range (70–180 mg/dL) to be defined as ‘good metabolic control’ (18). The other GV metrics include mean glucose, standard deviation (SD), coefficient of variation (CV), percentage of time spent in level 1 hypoglycemia (55–70 mg/dl) and level 2 hypoglycemia (< 55 mg/dl) (T Hypo), time in hyperglycemia level 1 (180–250 mg/dl) and level 2 (> 250 mg/dl) (T Hyper), number of hypoglycemia/hyperglycemia episodes with at least 15 min of duration, Mean Amplitude of Glycemic Excursions (MAGE), Mean of Daily Differences (MODD), the low blood glucose index (LBGI), the high blood glucose index (HBGI), Glucose Risk Assessment Diabetes Equation (GRADE), Average Daily Risk Range (ADRR), Glycemia Risk Index (GRI) (19), J index (a measure of quality of glycemic control based on the combination of information from the mean and SD calculated as 0.001 × (mean + SD)^2^, M100 (a measure of the stability of the glucose excursions in comparison with glucose value of 5.55 mmol/L [100 mg/dL]), time in tight range (TITR, percentage of time spent in glucose range 70-140mg/dl).

In this study, we used a selection of the available GV metrics.

### 2.4 Statistical analysis

Data were processed using Dell Statistica v.13. The Kolmogorov-Smirnov test was used to test the normality of the variables. Results are presented as means or medians for numerical variables and the number (%) for categorical variables. Student’s t-test and Mann-Whitney U test were used for comparisons of two groups based on the PSQI score, respectively. Categorical variables were compared using Pearson’s χ^2^ test. According to the NSF recommendations for sleep duration, we divided the study group into 3 subgroups: the group meeting the recommended sleep duration target of 7-9h (group T), the group sleeping less than 7h per night, L and sleeping more than 9h per night, M. Group comparisons were performed using one-way analysis of variance (ANOVA) for normally distributed data and the Kruskal–Wallis test for non-normally distributed data. *Post hoc* comparisons were adjusted using the Bonferroni correction. In the set of logistic regression models, we searched for glycemic predictors of sleeping in the target range. We used the univariable regression to identify significant predictors of sleeping in the target range and included them in a multivariable model.

A series of multivariable linear regression models was used to examine the relationships between sleeping in the target range and GV metrics after adjustment for possible confounding factors. The result of univariable analysis did not exclude predictors from entering them into multivariable analysis. Covariates included in multivariable models were based on the discussion in our clinical team. We decided that age and BMI, although not correlated with GV metrics in univariable models, were included because of possible clinical significance.

## 3 Results

The total of 155 participants with type 1 diabetes (73 men, 47%), mean (SD) aged 33 (9) years, median (IQR) diabetes duration 12 (8–20) years, were analyzed. At enrolment the mean (SD) HbA_1c_ was 57.2 (11.3) mmol/mol (7.4 [1.0]%). Sociodemographic and clinical characteristics of the study population are presented in **Table 1**. The mean (SD) sleep duration was 7.6 (1.4) h. No significant correlation was found between sleep duration and age, diabetes duration, HbA_1c_, BMI, and GV metrics when analyzing the study group in total. 78 participants (50.3%) met the NSF criteria of recommended sleep duration (target sleep group), 56 (36.1%) declared sleeping less than 7h, and 21 (13.6%) reported sleeping more than 9h.

**Table 1:** The sociodemographic and clinical characteristics of the study population and across predefined groups.

Characteristics	Study group, n=155	Sleeping less group, L, n=56	Target sleep group, T (7-9h per night), n=78	Sleeping more group, M, n=21	P-value
Age [years]	32.7 (8.9)	34.7 (8.0)†	32.9 (8.7)	26.8 (9.4)‡	**<0.001**
Sex, men/women [n (%)]	73 (47,1)	30 (53,6)	36 (46,1)	7 (33,3)	0.3”
Diabetes duration [years]	12 (8-20)	12.5 (9-20.5)	12 (8-18)	13 (8-17)	0.6
BMI [kg/m^2^]	25.5 (4.4)	25.5 (4.8)	25.5 (4.1)	25.1 (4.3)	0.8
HbA_1c_ [mmol/mol], IFCC	57.2 (11.3)	59.1 (9.7)	55.4 (11.7)	59.3 (13.1)	0.1
HbA_1c_ [%], DCCT	7.4 (1.0)	7.5 (0.9)	7.2 (1.1)	7.6 (1.2)	0.1
Sleep duration [h]	7.6 (1.4)	6.3 (0.7)*†	7.9 (0.5)	10.1 (0.8)‡	**<0.001**
PSQI Score [points]	6 (4-9)	7 (5-8)	6 (4-9)	7 (6-8)	0.5
PSQI Score>5 (poor sleep quality), [n(%)]	98 (63)	36 (64.3)	46 (59.0)	16 (76.2)	0.3”
Treatment regimen, [n, multiple insulin injections/insulin pump]	83/72	37/19	37/41	9/12	0.06”

Data are presented as means (SD), for normally distributed variables or medians (IQR) for non-normally distributed variables or n (% in group) for categorical variables. Group comparisons performed with one-way analysis of variance (ANOVA) for normally distributed data and the Kruskal–Wallis test for non-normally distributed data. *Post hoc* comparisons adjusted with the Bonferroni correction “Pearson’s χ2 test. *P<0.05 L vs T, †P<0.05 L vs M, ‡P<0.05 T vs M. PSQI, Pittsburgh Sleep Quality Index.

Statistically significant P-values (p<0.05) in bold.

Significant age differences were observed among the groups: participants sleeping <7 hours were older than those in the target sleep group, while participants sleeping >9 hours were younger than the target group (P<0.001).

Sleep quality was classified as poor (PSQI score>5) in 98 participants (63%), with a higher frequency among women (P = 0.04). No differences in GV metrics were observed according to sleep quality and no significant correlations were found between PSQI scores and GV metrics in the study group.

We observed significant differences in GV metrics among participants in the target sleep group compared with each other group, sleeping less or more – lower mean glucose, CV, GRI, HBGI, ADRR, GMI, MAGE, GRADE, M100, J index, and MODD, and higher TIR (**Table 2**). Only in the target sleep group the mean glycemic variability (%CV) was within the target of ≤36% as recommended by the International Consensus on Time in Range (18). Although significant differences were observed among the three compared groups in mean glucose, TIR, GRI, HBGI, GMI, and GRADE, the *post-hoc* analysis did not identify significant pairs of groups. No differences in low blood glucose metrics (LBGI), sleep quality, diabetes duration, HbA_1c_, or BMI were shown among groups. In multivariable logistic regression analysis sleeping in target range of 7-9h daily was associated with lower GV parameters, compared with sleeping less or more (combined groups not meeting NSF criteria): CV (OR 0.92 [95%CI, 0.86-0.97], P = 0.004), MAGE (OR 0.97 [95%CI, 0.96-0.99], P<0.001), and MODD (OR 0.95 [95%CI, 0.92-0.97], P = 0.005), after adjustment for age, sex and HbA_1c_ (**Table 3**).

**Table 2:** The comparison of GV indices across predefined sleep groups.

GV indices	Study group, n=155	Sleeping less group, L, n=56	Target sleep group, T (7-9h per night), n=78	Sleeping more group, M, n=21	P-value
Mean glucose [mg/dl]	167.0 (30)	172.2 (28.6)	161.2 (29)	174.6 (34.4)	**0.03**
CV [%]	37.1 (6.2)	38.6 (5.7)*	35.5 (6.3)	38.8 (6)	**0.02**
TIR [%]	60.4 (16.3)	57.7 (15.4)	63.5 (16.5)	56.1 (16.6)	**0.04**
TITR [%]	38.1 (14.9)	36.1 (13)	40.5 (16.2)	34.8 (13.5)	0.1
GRI	47.6 (20.8)	51.6 (20.7)	43.2 (20)	53.6 (21.2)	**0.02**
LBGI	0.66 (0.36-1.1)	0.64 (0.37-0.9)	0.69 (0.38-1.1)	0.58 (0.36-1.16)	0.8
HBGI	7.4 (5-11.7)	9.5 (5.3-13.2)	6.9 (4.6-9.4)	9.8 (5.6-11.9)	**0.02**
ADRR	41.5 (11.2)	44.5 (12.1)*	38.6 (10.3)	44.4 (9.1)	**0.01**
GMI [%]	7.3 (0.7)	7.4 (0.7)	7.2 (0.7)	7.5 (0.8)	**0.03**
MAGE	117.7 (31)	127.8 (32.8)*	108 (27.5)	126.8 (28.1)†	**0.001**
GRADE	9.5 (3.5)	10.2 (3.4)	8.8 (3.5)	10.5 (4)	**0.02**
M100	20.5 (12.7-35.1)	27.7 (14.1-40)*	18.2 (11.5-27)	27.5 (15.5-35.3)	**0.02**
J index	49.7 (38.5-68)	53.2 (41-74.6)*	46.5 (37.2-57.9)	59.1 (42.7-69.1)	**0.01**
AUC	158.4 (33.7)	163.4 (27)	155 (36.9)	157.9 (37.6)	0.06
MODD	62.7 (16.7)	68 (17.3)*	57.6 (14.5)	69.9 (18.2)†	**0.002**

Data are presented as means (SD) for normally distributed variables or medians (IQR) for non-normally distributed variables. Group comparisons performed with one-way analysis of variance (ANOVA) for normally distributed data and the Kruskal–Wallis test for non-normally distributed data. *Post hoc* comparisons adjusted with the Bonferroni correction *P<0.05 L vs T, †P<0.05 T vs M. SI conversion factors: to convert glucose to mmol/l, multiply by 0.0555. ADRR, Average Daily Risk Range; AUC, area under the curve; CV, coefficient of variation; GMI, Glucose Management Indicator; GRADE, Glucose Risk Assessment Diabetes Equation; GRI, Glycemia Risk Index; HBGI, high blood glucose index; LBGI, low blood glucose index; MAGE, Mean Amplitude of Glycemic Excursions; MODD, Mean of Daily Differences; TIR, time in range; TITR, time in tight range.

Statistically significant P-values (p<0.05) in bold.

**Table 3:** Results of the logistic regression analysis models of the possible independent glycemic predictors associated with sleeping in target range (7-9h).

	Model 1 unadjusted		Model 2 adjusted for covariates	
Predictors	OR (95% CI)	P-value	OR (95%CI)	P-value
Mean glucose [mg/dl]	0.98 (0.97-0.99)	**0.02**	0.99 (0.96-1.04)	0.9
CV [%]	0.91 (0.87-0.97)	**0.002**	0.92 (0.86-0.97)	**0.004**
TIR [%]	1.02 (1.00-1.05)	**0.02**	1.0 (0.99-1.06)	0.1
TITR [%]	1.02 (0.99-1.04)	0.051	1.0 (0.98-1.06)	0.3
GRI	0.98 (0.96-0.99)	**0.008**	0.98 (0.95-0.99)	**0.04**
LBGI	1.05 (0.68-1.62)	0.8	0.84 (0.51-1.39)	0.5
HBGI	0.91(0.85-0.97)	**0.009**	0.89 (0.79-0.99)	**0.04**
ADRR	0.95 (0.92-0.98)	**0.001**	0.95 (0.91-0.98)	**0.006**
GMI [%]	0.57 (0.35-0.9)	**0.02**	0.52 (0.24-1.14)	0.1
MAGE	0.98 (0.96-0.99)	**<0.001**	0.97 (0.96-0.99)	**<0.001**
GRADE	0.88 (0.8-0.97)	**0.01**	0.86 (0.73-1.01)	0.06
M100	0.97 (0.95-0.99)	**0.006**	0.96 (0.93-0.99)	**0.02**
J index	0.97 (0.96-0.99)	**0.003**	0.96 (0.94-0.99)	**0.01**
AUC	0.99 (0.98-1.01)	0.2	0.99 (0.98-1.01)	0.8
MODD	0.96 (0.94-0.98)	**<0.001**	0.95 (0.92-0.98)	**<0.001**

Model 1 is unadjusted. Model 2 is adjusted for covariates: age, sex, HbA_1c_. OR, odds ratio; 95%CI, 95% confidence interval. SI conversion factors: to convert glucose to mmol/l, multiply by 0.0555. ADRR, Average Daily Risk Range; AUC, area under the curve; CV, coefficient of variation; GMI, Glucose Management Indicator; GRADE, Glucose Risk Assessment Diabetes Equation; GRI, Glycemia Risk Index; HBGI, high blood glucose index; LBGI, low blood glucose index; MAGE; Mean Amplitude of Glycemic Excursions; MODD, Mean of Daily Differences; TIR, time in range; TITR, time in tight range.

Statistically significant P-values (p<0.05) in bold.

Multivariable linear regression was used to estimate the independent association between sleeping in the target range and glucose variability measures, after adjustment for age, sex, HbA_1c,_ and BMI. In those models sleeping in the target range was associated with lower CV (β=-0.24; R^2^ = 0.1, P = 0.003), lower GRI (β=-0.12; R^2^ = 0.49, P = 0.048), lower ADRR (β=-0.2; R^2^ = 0.38, P = 0.005), lower MAGE (β=-0.24; R^2^ = 0.41, P<0.001), lower M100 (β=-0.12; R^2^ = 0.63, P = 0.02), lower J index (β=-0.13; R^2^ = 0.64, P = 0.01), lower MODD (β=-0.25; R^2^ = 0.42, P<0.001). No associations of sleeping in the target range with TIR, GMI, and GRADE were observed (**Table 4**).

**Table 4:** Results of the multivariable linear regression models for target sleep as an independent predictor of selected GV indices.

Independent predictor	CV	GRI	ADRR	MAGE	M100	J-index	MODD
Target sleep (yes)	−0.24 (0.50) (P = 0.003)	−0.12 (1.30) (P = 0.048)	−0.20 (0.76) (P = 0.005)	−0.24 (2.05) (P < 0.001)	−0.12 (0.90) (P = 0.02)	−0.13 (1.00) (P= 0.01)	−0.25 (1.10) (P < 0.001)
Model R²	0.11		0.49	0.38	0.41	0.63	0.64

Models adjusted to age, sex, BMI, HbA1c. Results are presented as standardized coefficient (β) (SE) with an appropriate P-value for predictor and coefficient of determination (R^2^) for the whole model.

## 4 Discussion

Although the importance of adequate sleep as a fundamental biological process—crucial for maintaining physical health, cognitive performance, and emotional regulation—has been widely acknowledged and incorporated into general lifestyle recommendations for healthy living, many individuals still fail to achieve sufficient sleep duration or quality. In our study, only 50.3% of participants met the NSF criteria of recommended sleep duration time of 7-9h.

**Figure 2:**
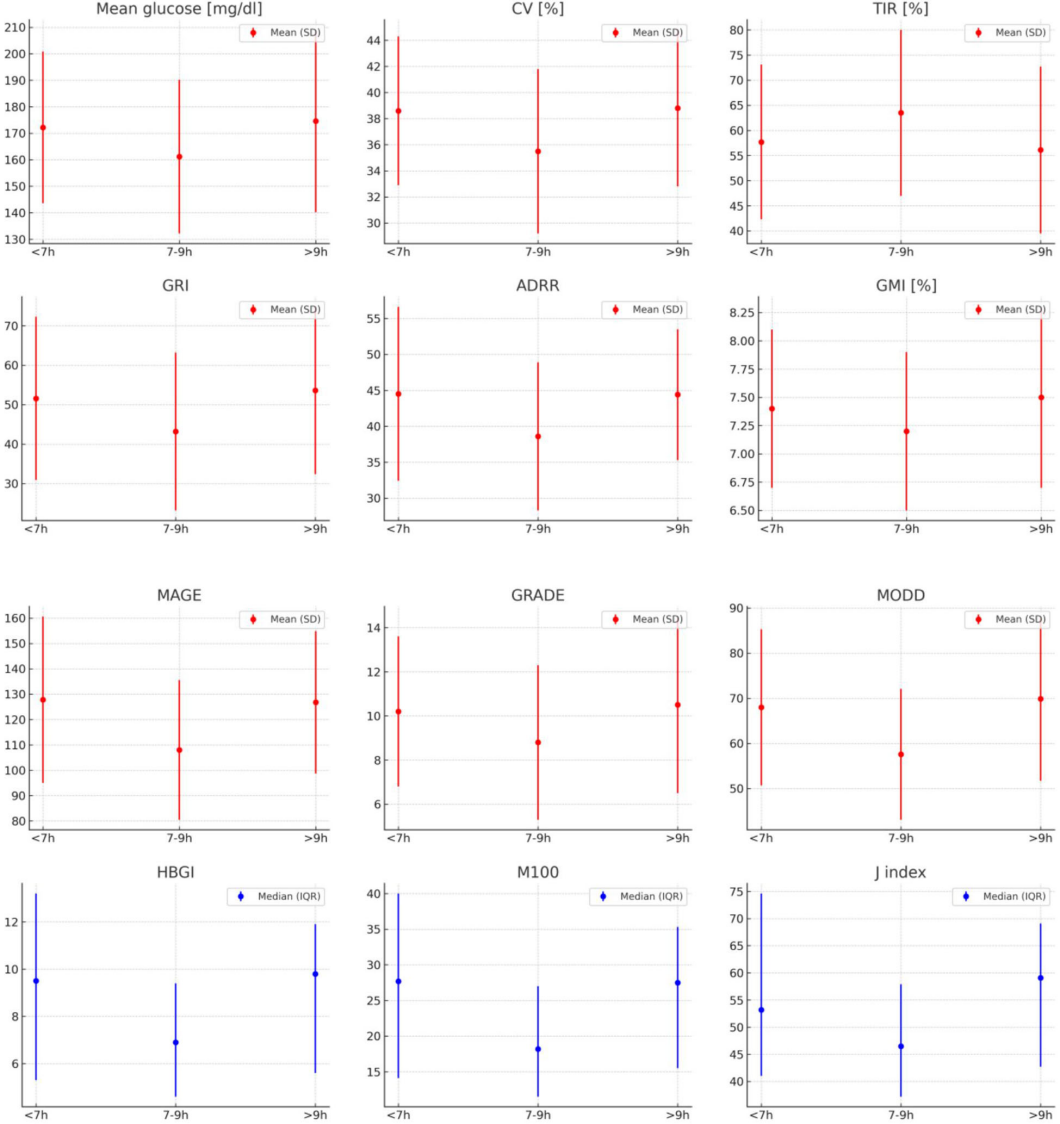
GV metrics in predefined sleeping groups. Mean (SD) in red, median (IQR) in blue.

The main finding of our study was that participants who slept within the target duration exhibited the lowest glycemic variability (GV) metrics (**Figure 2**). Although we did not observe any direct, statistically significant correlations between GV metrics and sleep duration or quality in the overall sample, we did find significant differences in multiple GV metrics when comparing the three predefined sleep duration groups. Similarly, Reutrakul S. et al. showed no associations between CGM-derived glycemic variability indices and sleep duration in 76 adults with type 1 diabetes and a mean sleep duration of 6.7 ± 0.85h (20). One possible explanation is provided by the current study: the associations between CGM-derived glycemic variability indices and sleep duration are non-linear, with the lowest glucose variability observed in the target 7–9 hour sleep group. A non-linear relationship in nature is not unusual and refers to a type of association between two variables where both low and high levels of one variable are associated with negative outcomes, while moderate levels are associated with more optimal or beneficial outcomes, e.g. BMI and mortality (21) or exercise intensity (both sedentary behavior and excessive high-intensity exercise can have negative health effects) (22). The non-linear relationship is also documented for sleep. Both short and long sleep duration are associated with an increased risk of all-cause mortality (23), hypertension (24), stroke (25), metabolic syndrome (26), cognitive decline (2), and major depressive episode or anxiety disorders (3). However, the question of causality remains to be answered, since short and long sleep durations might be risk factors, early markers, or a result of chronic clinical conditions (27, 28).

Interestingly, we found no differences according to sleep quality in the target sleep group compared to sleeping less or more. Also, there was no correlation of PSQI score with GV metrics across the study group. 63% of participants were bad-sleepers (PSQI>5). The prevalence of poor sleep or “bad sleepers” based on PSQI score among people with type 1 diabetes varies significantly across studies. The VARDIA Study, a multicentric cross-sectional study on sleep and type 1 diabetes, showed PSQI>5 in 156 (59.8%) of participants in the cohort of 315 adults (29) and no significant differences in GV measures assessed from 7-point self-monitoring of blood glucose (SMBG) between good- and bad-sleepers. However, a high CV% is associated with poor sleep quality. Botella-Serrano M. et al. reported poor sleep in 12 of 23 adults with type 1 diabetes, with sleep disturbances contributing most to the high PSQI score (sudden nocturnal awakenings or other reasons like heat, cold, pain, nightmares, snoring, coughing, or the need to urinate) (30). Bongiorno C et al. found altered sleep quality detected by PSQI questionnaire in 142 subjects (32.1% of 443 adults with autoimmune diabetes), while reduced total sleep time (TST) was observed in 177 patients (40.0% of the 443-cohort) (31). In the Italian cohort of 189 adults with type 1 diabetes treated with insulin pumps (122 participants had automated insulin delivery system, AID) 72% were bad-sleepers (PSQI>5) and no differences in proportion of bad-to good sleepers between participants using traditional continuous subcutaneous insulin infusion CSII vs AID were shown according to sleep quality (32). In our study, only 9 participants were active AID users (Medtronic MiniMed™ 780G with Guardian™ 4 Sensor). The issue of automatic insulin delivery and sleep quality/duration seems an interesting direction for future analyses, as there are not many studies on this topic, and the results are inconsistent according to the sleep outcomes. Polonsky WH et al. showed no significant change in reported sleep quality, but improved GV metrics among 115 adults with type 1 diabetes using AID for 3 months compared with multiple daily injections of insulin (33). One study showed even worsening of sleep quality after introducing AID for the overnight period; however, GV metrics improved (34).

In this study, we showed the lower GV metrics in people meeting the recommended 7-9h sleep. There is a need for further studies on this topic, as no simple explanation for this association exists, and the issue is multidimensional. We understand that there is no one-size-fits-all recommendation according to sleep duration, and some individual differences in the need for sleep exist. Chronotype is an example of individual sleep timing preference that can be measured using appropriate questionnaires (e.g., Horne-Ostberg Morningness-Eveningness Questionnaire, MEQ) and has a genetic background with a specific set of genes that might modulate core circadian rhythms or light-sensing pathways (35). Disruptions in the circadian rhythm, caused by irregular schedules, shift work, exposure to artificial light, and social jet lag, can lead to sleep disturbances and, as a consequence, to certain biochemical, cellular, hormonal, and metabolic disturbances. Sleep deprivation has been shown to suppress T-cell function, imbalance the release of proinflammatory cytokines (36), or diminish phagocytic and NADPH oxidase activity of neutrophils (37). Numerous human and animal studies have also demonstrated the influence of sleep factors on hormone levels (38). Sleep deprivation can cause alterations in growth hormone and prolactin concentrations (36). In addition, LAN (light exposure at night) in individuals with late habitual sleep timing, insomnia, or an evening chronotype can potentially suppress melatonin, increase circulating estrogen levels, and alter estrogen signaling pathways (39). Cortisol, the stress hormone, is produced during negative and positive experiences and is crucial in promoting alertness, focus, and energy. However, prolonged or excessive stress can have detrimental effects on health. High cortisol levels over an extended period can disrupt sleep patterns, impair cognitive function, weaken the immune system, and contribute to various health problems. An increase in morning serum cortisol levels has also been reported following sleep fragmentation, potentially contributing to morning insulin resistance (40). Sleep deprivation and fragmentation may lead to a shift in sympathovagal balance toward an increase in sympathetic nervous system activity, as reflected by lower heart rate variability (41), and is associated with disturbed metabolism of glucose (42). Increased sympathetic nervous system activity has inhibitory effects on insulin secretion and promotes insulin resistance and the development of the metabolic syndrome (43). Sleep restriction was shown to result in increased concentrations of circulating non-esterified (free) fatty acids (NEFA) during the nocturnal and early-morning hours in healthy men. The elevation in NEFA was related to prolonged nocturnal growth hormone secretion and higher early-morning noradrenaline levels and correlated with reduced insulin sensitivity after sleep restriction (44).

When comparing three different sleep duration groups, those who slept less than 7h per night were the oldest, which is in line with existing data for sleep ranges in different age groups (7). No direct correlation of sleep duration and HbA_1c_ or BMI was found, yet in our cohort mean (SD) BMI was 25.5 (4.4) kg/m^2,^ with only 22 participants with a BMI ≥ 30 kg/m^2^. There were no differences in HbA_1c_ or BMI according to sleep quality based on PSQI score, respectively. The data from a cumulative total of 5,172,710 participants collected from 153 studies showed the association of short sleep with many health complications, including obesity (RR, 1.38, 95%CI, 1.25-1.53) (45). Makhdom AE et al. in the prospective SLEEP T2D study have recently shown a negative correlation of BMI (r = -0.27, P < 0.001) and waist circumference (r = -0.25, P = 0.001) with sleep duration in a cohort of adults with T2D. In that study, short sleep at baseline was associated with a 5% or more gain in BMI in a median follow-up of 26.5 months (46). The growing evidence supports the issue of optimal sleep as a key element in maintaining a healthy body weight (47).

### 4.1 Limitations

The inaccuracy of self-reported sleep duration and quality could not be eliminated as sleep assessment was based on the PSQI questionnaire, not on direct actigraphy or polysomnography, which allow for objective measurements of certain sleep variables and reflect general sleep quality. Studies that collected sleep information via self-report may present an overestimated sleep duration compared to objective measurements (48).

Therefore, a more accurate evaluation of sleep duration is a key issue that should be addressed in future studies. In addition, a limitation of our study is its cross-sectional design. Prospective longitudinal observations would provide more robust insights.

As the data on sleep quality and duration were based on self-reported surveys and not directly measured, we could not perform analyses of the regularity and repeatability of individual sleep patterns. Some studies present sleep regularity as a more important factor influencing wellbeing and a stronger predictor of mortality risk than sleep duration itself (49), and thus it might also be an important factor influencing GV (50).

### 4.2 Conclusions

This study shows significant differences in glycemic variability metrics according to sleep duration. Adults with type 1 diabetes who sleep recommended 7–9 hours per night present lower glycemic variability compared with sleeping less or more.

The essential role of sleep in overall health is increasingly recognized, and our study adds new insights in both clinical and public health contexts according to the associations between sleep and glycemic variability in type 1 diabetes.

## Data Availability

The raw data supporting the conclusions of this article will be made available by the authors, without undue reservation.
